# The impact of tropodithietic acid on microbial physiology under varying culture complexities

**DOI:** 10.1128/msphere.00138-25

**Published:** 2025-07-18

**Authors:** Olesia Shlakhter, Sergey Malitsky, Einat Segev

**Affiliations:** 1 Department of Plant and Environmental Sciences, Weizmann Institute of Science, Rehovot, Israel; 2 Department of Life Sciences Core Facilities, Weizmann Institute of Science, Rehovot, Israel; Clemson University, Clemson, South Carolina, USA

**Keywords:** microbial interactions, algal-bacterial interactions, tropodithietic acid, bacterial physiology, marine bacteria

## Abstract

Understanding marine bacterial physiology under environmentally relevant conditions requires the study of biotic interactions across systems of varying complexities. Here, we examine how the capability of *Phaeobacter inhibens* bacteria to produce tropodithietic acid (TDA), a secondary metabolite, influences microbial physiology and interactions. Our systematic approach, which includes progressing from bacterial monocultures to co-cultures and tri-cultures involving algal hosts, allows us to evaluate the impact of the *tdaB* gene and the TDA metabolite on microbial interactions. Our findings show that deleting the *tdaB* gene resulted in no detectable TDA production and affected bacteria-bacteria interactions in co-culture but not in tri-cultures with the algal host. Additionally, our data reveal that algal death was delayed in cultures containing *P. inhibens* Δ*tdaB* mutants compared to those with wild-type bacteria, although no TDA was detected in these tri-cultures. The findings of our study highlight the importance of microbial complexity in the study of bacterial physiology and point to the understudied role of TDA in microbial interactions.

**IMPORTANCE:**

Laboratory model systems enable controlled studies of marine microbial processes; however, the microbial complexity of the culture can influence the outcome. In this study, we employ a systematic approach to assess the impact of the bacterial ability to produce the antibiotic TDA in laboratory cultures with varying microbial complexities (from bacterial monocultures to bacterial co-cultures and algal-bacterial tri-cultures). Our findings demonstrate altered effects of the *tdaB* gene deletion with increasing microbial complexity, showing distinct impacts on microbial fitness. Since antibiotics like TDA mediate microbial interactions, it is important to examine them within ecologically relevant model systems that reflect inter- and intra-trophic interactions, including bacteria-bacteria and algae-bacteria relationships. Overall, our study highlights the importance of accounting for culture complexity when designing laboratory experiments to investigate microbial interactions and the compounds that mediate them.

## INTRODUCTION

In recent years, research has increasingly concentrated on understanding marine bacterial physiology under environmentally relevant conditions. One key factor influencing microbial physiology is biotic interactions, which are often simulated in the lab through the microbial complexity of the cultures. For instance, bacterial physiology has been shown to be affected by various algal metabolites, as evidenced by studies on algal-bacterial cultures. Algal-driven bacterial responses include accelerated bacterial growth (1), formation of a bacterial extracellular matrix (2), enhanced bacterial conjugation (3), and increased synthesis of secondary metabolites (4). Additionally, bacteria grown in the presence of other bacterial species have demonstrated altered physiology, such as the production of novel secondary metabolites (5) or increased antibiotic production (6).

Notably, findings from cultures with low complexity that have been reassessed in more complex systems occasionally revealed different outcomes. For example, the pathogenicity of *Phaeobacter inhibens* bacteria toward *Emiliania huxleyi* (also known as *Gephyrocapsa huxleyi* [7]) algae was initially observed in algal-bacterial co-cultures (8–11). However, when this pathogenic behavior was examined in a community with additional bacteria, it was found that the pathogenicity did not manifest (12). It was also demonstrated that the effect of *P. inhibens* on host-associated microbiomes varies, depending on the complexity and composition of the existing microbiome (13). Therefore, understanding microbial physiology in laboratory models should account for the impact of microbial complexity within the system.

Here, we aimed to evaluate how the bacterial capability to encode the synthesis of a secondary metabolite influences microbial physiology under varying microbial complexities.

*P. inhibens* bacteria produce the secondary metabolite tropodithietic acid (TDA), which can act as an antibiotic against various organisms such as the fish pathogenic bacteria *Vibrio anguillarum* (14, 15). The synthesis of TDA can be completely abolished by deletion of the *tdaB* gene (16). *P. inhibens* bacteria are commonly found in algal blooms, where they interact with various co-occurring bacteria as well as with their algal hosts (11). To study how the ability of *P. inhibens* to produce TDA affects microbial physiology within different contexts of microbial complexity, we established both a co-cultivation system of *P. inhibens* with *Dinoroseobacter shibae* bacteria and a tri-cultivation system with the addition of the *Emiliania huxleyi* algal host. We incorporated either wild-type (WT) *P. inhibens* or *tdaB-*deleted mutants (Δ*tdaB*) into these co- and tri-cultures to assess the relationship between the *tdaB* gene, microbial physiology, and microbial complexity. Our findings reveal the importance of microbial complexity in the study of bacterial physiology and highlight the role of the *tdaB* gene in both bacterial-bacterial and algal-bacterial interactions.

## RESULTS

### Establishing experimental systems to study the impact of TDA on microbial interactions

**Fig 1:**
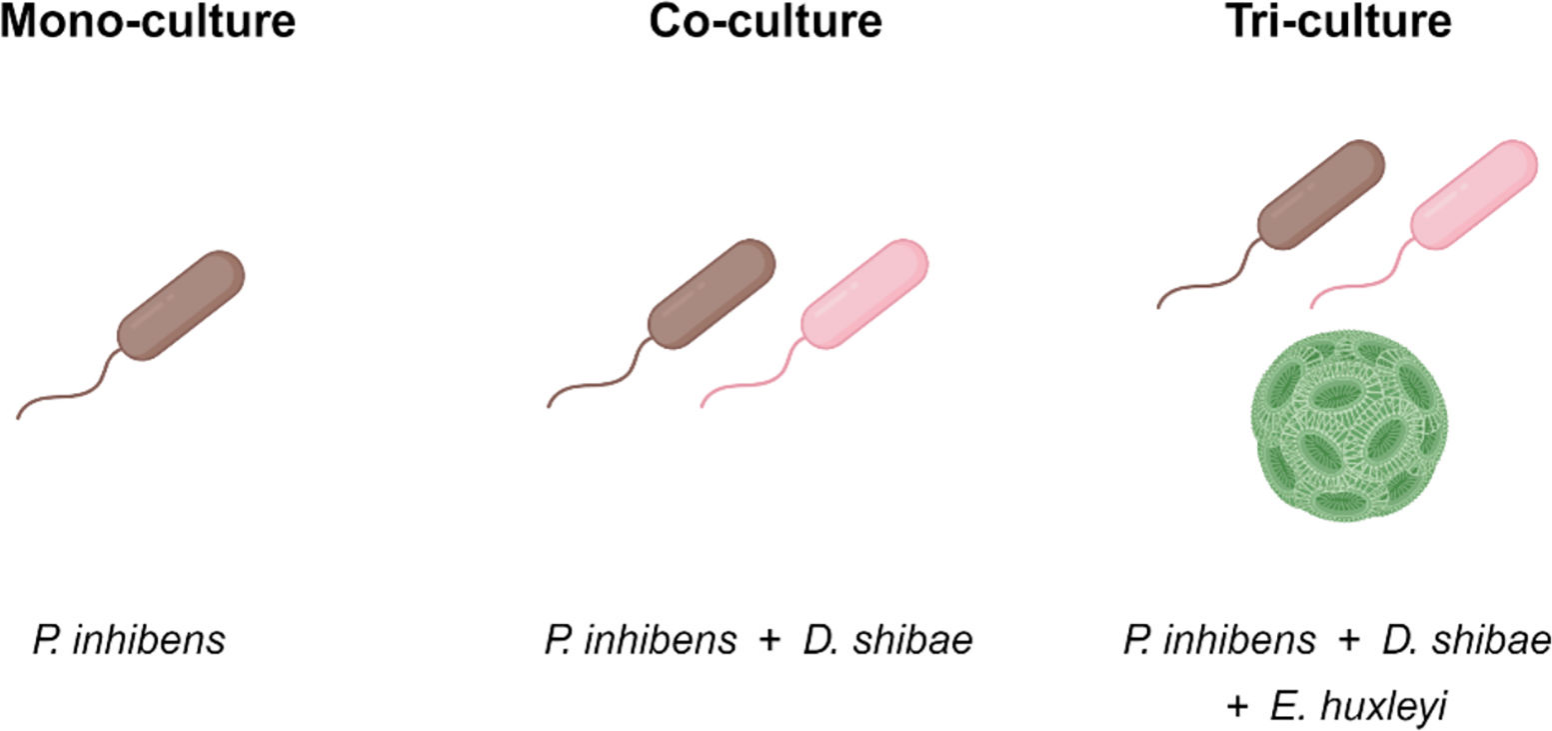
Schematic representation of the microbial cultures used in this study. Monocultures contain pure cultures of *P. inhibens* DSM 17395 wild-type (WT) or Δ*tdaB* mutant bacteria (as indicated), in CNS medium (see Materials and Methods) with 5.5 mM succinate as a sole carbon source. Co-cultures contain *P. inhibens* bacteria (WT or Δ*tdaB*, as indicated) with *D. shibae* DFL-12 bacteria in CNS medium containing 5.5 mM succinate as a sole carbon source. Tri-cultures contain *P. inhibens* bacteria (WT or Δ*tdaB*, as indicated), *D. shibae* bacteria, and *E. huxleyi* algae in synthetic seawater (see Materials and Methods) with no added carbon source.

To investigate the impact of TDA on interactions between *P. inhibens* and other microorganisms, we established three experimental systems with varying microbial complexities (Fig. 1). Each system included either WT *P. inhibens* or a mutant lacking the *tdaB* gene (Δ*tdaB*), essential for TDA production. Absence of TDA production in the Δ*tdaB* mutant was confirmed by ultra-performance liquid chromatography (UPLC-MS) (Table 1). The simplest system was a monoculture of *P. inhibens* (WT or Δ*tdaB*), grown in a defined medium with succinate as the sole carbon source (Fig. 1). In these cultures, cell growth was monitored over time by OD_600_ measurements (see Materials and Methods). To increase complexity, co-cultures were grown, consisting of *P. inhibens* (WT or Δ*tdaB*) with *D. shibae*, a bacterial species that naturally co-exists with *P. inhibens* during algal blooms of *E. huxleyi* (Fig. 1). Co-cultures were grown in defined medium with succinate as the sole carbon source, and selective plates were used for species-specific colony-forming unit (CFU) counts (see Materials and Methods). The most complex system was a tri-culture of *P. inhibens* (WT or Δ*tdaB*), *D. shibae*, and their algal host, *E. huxleyi* (Fig. 1). Tri-cultures were cultivated in artificial seawater without external carbon sources, compelling bacteria to depend on organic carbon from algal exudates. In tri-cultures, bacterial CFU counts were obtained using species-specific selective plates, complemented with a second independent method of quantitative PCR (qPCR) to validate the results (see Materials and Methods).

**TABLE 1:** Extracellular TDA levels in cultures with WT or Δ*tdaB P. inhibens*^*a*^

Culture type	*P. inhibens* WT		*P. inhibens* Δ*tdaB*	
	TDA (ng/mL)	Bacterial numbers (CFU/mL)	TDA (ng/mL)	Bacterial numbers (CFU/mL)
Monocultures	2,467 (± 362)	23.6 (±0.3) × 10^6^	ND^*b*^	25.4 (±0.5) × 10^6^
Co-cultures	1,080 (± 881)	22.5 (±6.5) × 10^6^	ND	26.3 (±8.5) × 10^6^
Tri-cultures (day 4)	ND	4 (±1) × 10^3^	ND	2 (±0.8) × 10^3^
Tri-cultures (day 7)	ND	2.7 (±0.7) × 10^6^	ND	2.3 (±0.7) × 10^6^

^*a*^ All cultures were sampled at the stationary phase (24 h for mono- and co-cultures, day 7 for tri-cultures), and all 30 mL of the filtrate was used for the analysis. In addition, tri-cultures were also sampled at the exponential phase (day 4) to test for potential growth phase effects on TDA production. Data were acquired via ultra-performance liquid chromatography-mass spectrometry (see Materials and Methods) using four biological replicates. Values are presented (in ng/mL) as mean (±standard deviation). Bacterial concentrations for corresponding time points are taken from Fig. 2 to 4 and presented as mean (±standard deviation) for mono- and co-cultures and as mean (±standard error) for tri-cultures.

^*b*^ ND, not detected.

**Fig 2:**
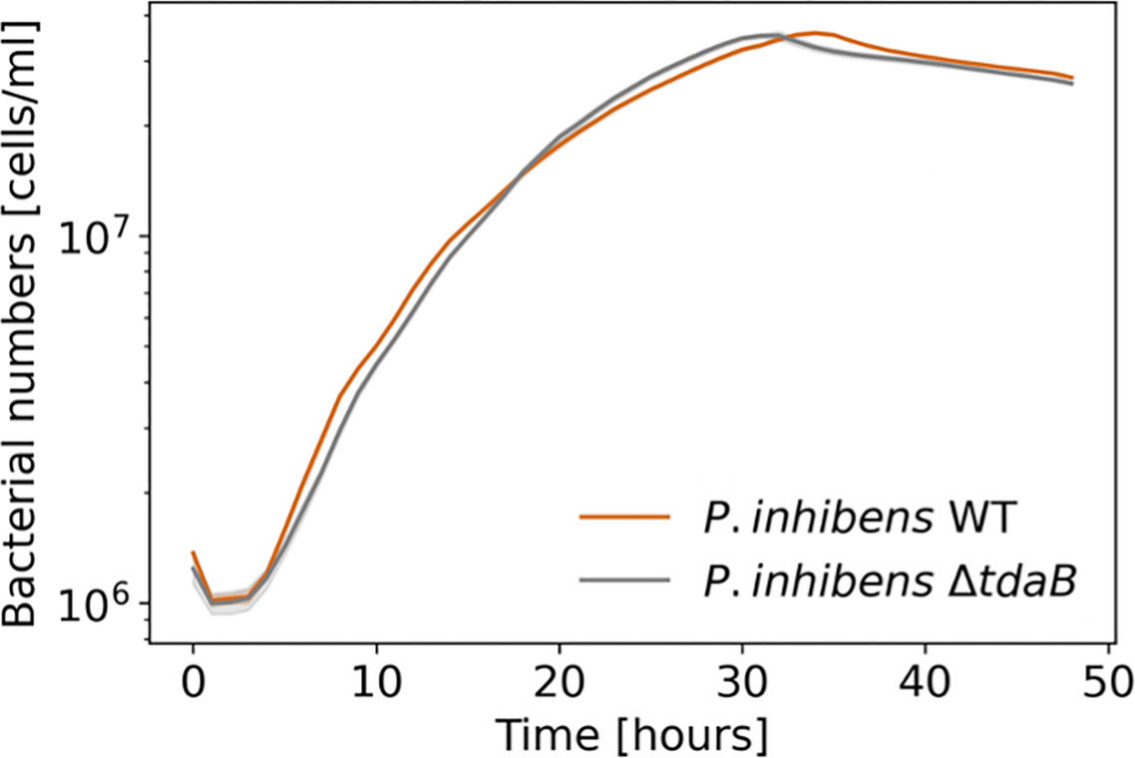
Deletion of the *tdaB* gene does not affect the growth of *P. inhibens* bacteria in monoculture. Growth of *P. inhibens* WT (brown) and Δ*tdaB* mutant (gray) bacteria in CNS medium containing succinate as a sole carbon source. Bacterial growth was monitored using OD_600_, and values were converted to cells per milliliter (see Materials and Methods). Each line represents the mean of four biological replicates, and shaded areas indicate the standard deviation.

The growth curves of monocultures were comparable between the WT and Δ*tdaB* strains of *P. inhibens* (Fig. 2), in agreement with previously published data on another *Phaeobacter* sp. (17). The comparable growth of WT and mutant *P. inhibens* strains in monocultures provides a baseline for assessing the impact of TDA on microbial interactions.

### Deletion of the *tdaB* gene impacts the fitness of *D. shibae* in co-cultures with *P. inhibens*

**Fig 3:**
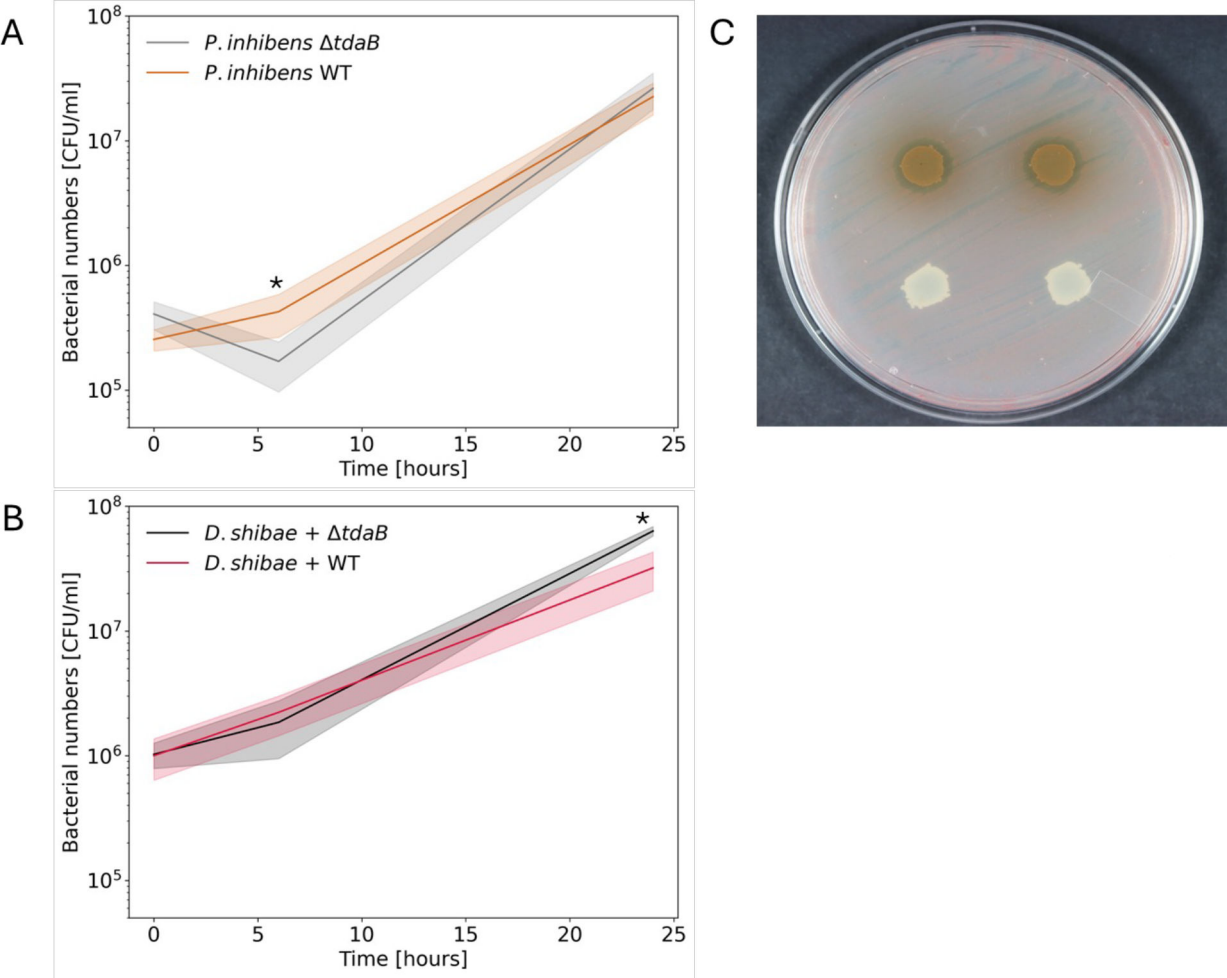
Deletion of the *tdaB* gene in *P. inhibens* increases the fitness of *D. shibae* bacteria in co-cultures. (**A**) Growth dynamics of *P. inhibens* WT (brown) and Δ*tdaB* (gray) bacteria, and (**B**) *D. shibae* bacteria grown with *P. inhibens* WT (pink) or Δ*tdaB* (black) in co-culture with CNS medium containing 5.5 mM succinate as a sole carbon source. The initial inoculum was 2.5 × 10^5^, 4 × 10^5^, and 10 × 10^5^ cells/mL for *P. inhibens* WT, *P. inhibens* Δ*tdaB,* and *D. shibae*, respectively (see Materials and Methods). Lines represent the mean value of four biological replicates, and the shaded area corresponds to the standard deviation. *P < 0.05 using a two-tailed *t*-test on log-transformed values. (C) A lawn of *D. shibae* was plated on an MB plate and spotted with 5 µL of *P. inhibens* WT (upper, brown colonies) and Δ*tdaB* mutant (lower, white colonies) cultures. Plates were incubated for 72 h at 30°C. Deletion of the *tdaB* gene resulted in loss of pigmentation as previously described (18) and no visible inhibition zone, characteristic of tropodithietic acid secretion (16).

To evaluate the impact of TDA on the interaction of *P. inhibens* and *D. shibae* bacteria, we first tested the susceptibility of *D. shibae* to TDA. We therefore plated a *D. shibae* lawn on marine broth (MB) agar plates and spotted *P. inhibens* WT and Δ*tdaB* cultures on top of the lawn. If *D. shibae* bacteria are killed or their growth is inhibited by TDA, a clear zone should be visible around colonies of WT *P. inhibens* bacteria but not around colonies of the Δ*tdaB* strain. Our results show that WT *P. inhibens* colonies exhibit the typical brown pigmentation associated with TDA production (16, 18), and a clear zone around the colonies was evident (Fig. 3C, top). In contrast, colonies of Δ*tdaB P. inhibens* lost their pigmentation, as previously described (18), and had no visible clear zone (Fig. 3C, bottom). These results confirmed that *D. shibae* is indeed susceptible to TDA produced by *P. inhibens* and that the Δ*tdaB P. inhibens* strain is perturbed in TDA production, as shown earlier via UPLC-MS (Table 1).

Next, we examined whether TDA production affects bacterial fitness in bacterial co-cultures. In liquid cultures, secondary metabolites (including TDA) are often produced by bacteria during later stages of growth as a result of nutrient limitations (19). Therefore, bacterial abundance in co-cultures was examined with an emphasis on differences after 24 h of growth, which corresponds to the transition between the late exponential and early stationary growth phases for both bacterial species (Fig. 2; Fig. S1). Indeed, TDA was detected after 24 h in co-cultures with WT but not Δ*tdaB P. inhibens* (Table 1). Our results show that *D. shibae* gains a slight increase in fitness after 24 h of cultivation in co-cultures with the *P. inhibens* Δ*tdaB* mutant compared to WT (Fig. 3B), while *P. inhibens* growth is comparable between strains (Fig. 3A).

### Deletion of the *tdaB* gene does not affect the fitness of *P. inhibens* or *D. shibae* bacteria in tri-cultures

In the marine environment, various heterotrophic bacteria rely on their algal hosts as a source of organic carbon (20, 21). Given the impact of *tdaB* deletion on the bacterial fitness in co-cultures when succinate is provided as the sole carbon source, we aimed to determine whether this alteration in fitness is also evident when *P. inhibens* and *D. shibae* rely on their algal host.

**Fig 4:**
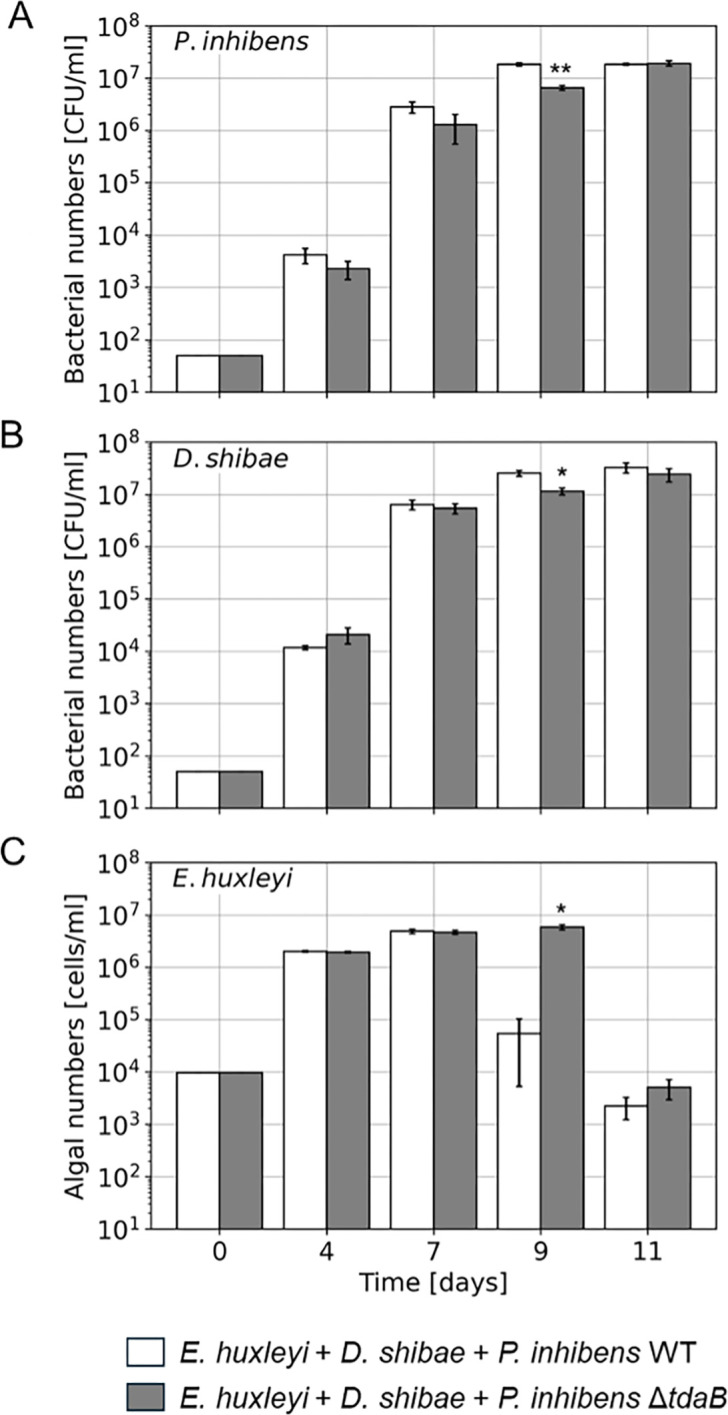
The deletion of the *tdaB* gene delays the pathogenicity of *P. inhibens* toward *E. huxleyi* algae in the context of tri-cultures. (**A**) Growth of *P. inhibens* WT bacteria (white bars) and Δ*tdaB* mutant bacteria (gray bars) in tri-cultures. (**B**) Growth of *D. shibae* bacteria in tri-cultures with *P. inhibens* WT strain (white bars) or the Δ*tdaB* mutant strain (gray bars). (**C**) Growth of *E. huxleyi* algae in tri-cultures with *D. shibae* bacteria and *P. inhibens* WT strain (white bars) or the Δ*tdaB* mutant strain (gray bars). Bacterial growth was monitored using selective plates, and algal growth was tracked using flow cytometry (see Materials and Methods). The differences in growth between cultures were evident at day 9 for all species. Bars represent mean values of three biological replicates. Error bars indicate standard errors. *P < 0.05, **P < 0.005 using a two-tailed *t*-test on log-transformed values.

Our data show that in tri-culture, bacterial final yields of both *P. inhibens* and *D. shibae* on day 11 were not affected by the *tdaB* gene deletion (Fig. 4A and B). We observed no significant differences in the final yields of WT and Δ*tdaB P. inhibens* bacteria in tri-cultures or in the final yield of *D. shibae* bacteria. Because *P. inhibens* bacteria attach and aggregate, possibly affecting CFU counts, we further validated bacterial abundance using qPCR to quantify DNA copy numbers on day 8 of cultivation. Bacterial numbers according to CFUs and qPCR were consistent, showing no significant differences in the cell numbers of WT and Δ*tdaB P. inhibens* or *D. shibae* bacteria in tri-cultures on day 8 (Fig. S2). Additionally, no TDA was detected in tri-cultures with WT and Δ*tdaB P. inhibens* (Table 1). These results align with previous studies that showed only a minor impact of TDA on the bacterial partners of diatom microalgae (22).

However, an interesting exception was noted on day 9; both *P. inhibens* and *D. shibae* showed decreased growth in tri-cultures containing the *ΔtdaB* strain. Since both bacterial species were similarly affected, it is difficult to attribute this decrease to a growth disadvantage specific to the absence of TDA. Instead, as discussed below, the reduced growth of both bacterial species appears to reflect changes in the algal host population dynamics.

### Deletion of the *tdaB* gene affects the pathogenicity of *P. inhibens* toward the algal host

*P. inhibens* bacteria display pathogenic behavior toward *E. huxleyi* algae, leading to algal death (10, 11). Our data show that deletion of the *tdaB* gene resulted in delayed pathogenicity of *P. inhibens* toward *E. huxleyi* both in tri-cultures (Fig. 4C) and in cultures of only *P. inhibens* with *E. huxleyi* (Fig. S3). This delay suggests that the expression of the *tdaB* gene may impact the pathogenicity of *P. inhibens* and could influence algal-bacterial interactions. Although the exact relationship between bacterial TDA production and algal death is not yet fully understood, the absence of the *tdaB* gene seems to delay algal death, thereby affecting the availability of algal-derived organic carbon for the associated bacteria. In tri-cultures with WT *P. inhibens*, bacterial growth of both *P. inhibens* and *D. shibae* exceeds densities of 10^7^ cells/mL upon algal death. In contrast, when algal death is delayed in tri-cultures with the Δ*tdaB* strain, bacterial growth of both bacterial species remains below 10^7^ cells/mL, and only following the delayed algal death, the density of the cultures exceeds 10^7^ cells/mL. In conclusion, the *tdaB* gene appears to play an overlooked role in both bacterial-bacterial and algal-bacterial interactions.

## DISCUSSION

### The importance of culture complexity in laboratory studies of microbial physiology

Secondary metabolites, especially antibiotics, shape microbial interactions (23). In the marine environment, host-associated bacteria produce a wide range of antibiotics, as demonstrated by bacteria from the algal microbiome (24) and sponge microbiome (25, 26). In turn, antibiotic production is significantly impacted by interactions among bacteria. For example, marine bacteria from the *Streptomyces tenjimariensis* sp. produce the antibiotic istamycin to outcompete susceptible bacterial species (27). Bacterial interactions can also inhibit antibiotic production, as reported for the coral-associated actinobacterium *Agroccocus* that impaired the ability of another *Streptomyces* actinobacterium to produce antibiotics (28).

Therefore, when studying the impact of secondary metabolites on microbial physiology in laboratory model systems, it is important to consider the potential influence of the microbial complexity within the system. In this study, we examined the impact of deleting the *tdaB* gene, which is essential for the production of the TDA antibiotic in *P. inhibens*, on interactions with ecologically relevant partners. Cultures with varying levels of microbial complexity—from monocultures of *P. inhibens* to co-cultures with *D. shibae* bacteria and tri-cultures with both bacteria and *E. huxleyi* algae—allowed us to evaluate the influence of the *tdaB* deletion on these interactions.

### TDA production in tri-cultures

Although no TDA was detected in the tri-cultures with the *P. inhibens* WT strain (Table 1), the deletion of the *tdaB* gene did impact the algal-bacterial interaction, resulting in delayed algal death. Several factors could explain this observation.

First, *P. inhibens* is known to attach to algal cells and exchange metabolites in close proximity to the host (11). The immediate environment surrounding the algal cell, referred to as the boundary layer, is chemically distinct from the bulk solution (29). This localized area may contain higher concentrations of TDA than the bulk solution, where TDA levels may be below the detection limit of our study (0.25 ng/mL).

Another possibility is that TDA itself is not produced in tri-cultures. Instead, deletion of the *tdaB* gene may impact other metabolic pathways. For instance, deletion of the *tdaE* gene alters the exometabolome of *P. inhibens*, affecting the production of vitamins, amino acids, siderophore components, and other metabolites (30). Furthermore, the *tda* operon is involved in the production of roseobacticides, molecules that induce algal death (31). Therefore, deletion of the *tdaB* gene may affect roseobacticide production and delay the pathogenicity of *P. inhibens* toward algae.

### The impact of TDA on bacterial interactions depends on the culture complexity

The influence of the *tdaB* gene deletion on bacterial interactions appears to depend on the culture complexity. In our study, deleting the *tdaB* gene in *P. inhibens* affected its interaction with *D. shibae* in co-culture (Fig. 3), but not in tri-culture when the algal host *E. huxleyi* was present (Fig. 4). A key difference between co-cultures and tri-cultures that should be noted is the composition and flux of the bacterial nutrient supply. In co-cultures, bacteria receive a defined medium with succinate as a sole carbon source. Essential nutrients are supplied as fresh medium at the onset of the experiment and are gradually depleted as bacteria consume them during growth. Under these conditions, competition between *P. inhibens* and *D. shibae* is likely to occur, consequently driving the expression of the *P. inhibens tdaB* to inhibit *D. shibae*. However, in tri-cultures with the algal host, bacteria experience a continuous flux of algal secreted compounds—termed algal exudates—consisting of a complex mixture of nutrients and biomolecules (32, 33). The variety of compounds in algal exudates can allow different bacteria to consume different resources, thereby driving niche partitioning and preventing competition (34–36). Moreover, algal exudates can impact the expression of various biosynthetic pathways in *P. inhibens* (4, 11, 37). Therefore, it is possible that *tdaB* expression is regulated differently in co-cultures versus tri-cultures, resulting in different levels of TDA. Interestingly, a previous study demonstrated that *P. inhibens* bacteria actively express *tdaB* and *tdaC* genes when part of the bacterial population associated with the green microalga *Tetraselmis suecica* (38). These findings suggest that the identity of the algal host, or the co-occurring bacterial species, impacts the regulation of TDA production.

### A potential link between TDA and bacterial pathogenicity

Bacterial antibiotics are widely studied in the context of bacterial interactions (39, 40), but their role in algal-bacterial associations is less understood. In the current study, the ability of *P. inhibens* to produce TDA had an impact on the algal host. *P. inhibens* WT bacteria induce algal death, a phenomenon that was previously studied by us (8, 11, 12) and by others (9, 10, 41). Our data now show that algal death was delayed in cultures with *P. inhibens* Δ*tdaB* mutants compared to WT bacteria. The delay in algal death occurred in both tri-cultures (Fig. 4) and in cultures with *E. huxleyi* and *P. inhibens* Δ*tdaB* (Fig. S3). Whether TDA is directly involved in bacterial pathogenicity, or whether the TDA impact on bacterial physiology has an indirect influence on pathogenicity is yet to be understood.

To conclude, our study demonstrates the importance of microbial complexity in the study of bacterial physiology and highlights gaps in our understanding of the various roles that secondary metabolites play in microbial interactions.

## MATERIALS AND METHODS

### Strains and growth conditions

The algal axenic strain *Emiliania huxleyi* CCMP3266 (also known as *Gephyrocapsa huxleyi* [7]) was purchased from the National Center for Marine Algae and Microbiota (Bigelow Laboratory for Ocean Sciences, Maine, USA). Algae were cultivated in artificial sea water (ASW) containing L1-Si supplements (Na_2_SiO_3_-omitted L1 medium according to Guillard and Hargraves [42]). Cultures were kept in 18°C under a light/dark cycle of 16 h/8 h and illumination intensity of 130 µmol photons/m^2^/s. The axenic state of algal cultures was monitored weekly by plating and under the microscope.

The bacterial strain *Phaeobacter inhibens* DSM 17395 was purchased from the German Collection of Microorganism and Cell Cultures (DSMZ, Braunschweig, Germany). Bacteria were plated from glycerol stocks (stored at −80°C) on one-half yeast extract-tryptone-sea salts (YTSS) agar plates (2 g yeast extract [Sigma-Aldrich, USA], 1.25 g tryptone [Sigma-Aldrich], 20 g sea salt [Sigma-Aldrich], and 16 g agar/L [BD Difco, USA]), and after 48 h of incubation at 30°C, single colonies were inoculated into liquid medium supplemented with carbon, nitrogen and sulfur (CNS) for a pre-culture (5.5 mM glucose [Sigma-Aldrich], 5 mM NH_4_Cl [Sigma-Aldrich], and 33 mM NaSO_4_ [Thermo Fisher Scientific, UK]) and diluted in ASW. Pre-cultures were cultivated in 30°C in the dark with 130 rpm shaking for at least 48 h to reach the stationary phase.

The bacterial strain *Dinoroseobater shibae* DFL-12 (DSM 16493) was received from the lab of Prof. Irene Wagner Dobler (DSMZ). Bacteria were plated from glycerol stocks on MB agar plates (37.4 g MB powder [BD Difco, USA] and 16 g agar/L [BD Difco]). Single colonies were inoculated into liquid CNS medium for a pre-culture (5.5 mM sodium succinate [Sigma-Aldrich], 5 mM NH_4_Cl, and 33 mM NaSO_4_) and diluted in ASW and cultivated at 30°C in the dark with 130 rpm shaking for at least 48 h.

The mutant strain *Phaeobacter inhibens* DSM 17395 Δ*tdaB* (*tdaB*::Gm^R^) was generated in the current study (see below) and cultivated under the same conditions used for the WT *P. inhibens* DSM 17395 strain, with the addition of 30 µg/mL gentamicin.

### Deletion of the *tdaB* gene in *P. inhibens*

To generate the *tdaB* gene deletion mutant of *P. inhibens* DSM 17395, the mutant strain ES150 was generated by replacing the gene PGA1_262p00970 (new tag PGA1_RS18610) with a gentamicin resistance cassette. Regions of approximately 1,000 bp upstream and downstream of the gene were PCR-amplified using primers 1021, 1022, 1023, and 1024, respectively (Table 2). The amplified fragments were assembled and cloned into the pDN18 vector (Table 3) using restriction-free cloning (43, 44). The resulting plasmid, pES1 (Table 3), was introduced into competent *P. inhibens* bacteria by electroporation. Preparations of competent cells were performed as previously described (45), with slight modifications. Briefly, cells were grown to an OD_600_ of 0.7 in one-half YTSS with 40 g/L of sea salt (also termed full salt medium). Bacteria were washed three times using 10% (vol/vol) ice-cold glycerol, centrifuged each time for 5 min at 4°C at 6,200 × *g*. The competent cells were subsequently adjusted to an OD_600_ of 20 using 10% (vol/vol) ice-cold glycerol, frozen in liquid nitrogen, and stored at −80°C. Electroporation was conducted with 300 µL aliquots of electrocompetent cells using 10 µg of DNA in a 2 mm cuvette at 2.5 V, followed by 4 h recovery in one-half YTSS with full sea salt concentration. The transformed cells were then plated on one-half YTSS plates containing 30 µg/mL gentamicin, and resistant colonies were validated by PCR (Table 2, primers 1025 and 1026) and DNA sequencing.

**TABLE 2:** Primers used in this study for mutagenesis

Primer	F/R^*a*^	Sequence	Comment
1021	F	GCAGGCTCCGAATTCGCCCTTAATCAAAACGATGGAAGACACGC	Amplification of an upstream region
1022	R	CGAACCGAACAGGCTTATGTCAACTGGGCGCTCCATATTGAAATTATAGG	Amplification of an upstream region
1023	F	GATATCGACCCAAGTACCGCCACCTAACAGTGGCCCTCATGGGTAGTC	Amplification of a downstream region
1024	R	CTTTGTACAAGAAAGCTGGGTCGAATTCGCCCTTGACGTTGCTGGAGACAAAGCG	Amplification of a downstream region
1025	F	GTGCCATTGTATAGCGGTGC	Validation of the mutation
1026	R	TGGTAAATCCCATCGGAACG	Validation of the mutation

^*a*^ F, forward; R, reverse.

**TABLE 3:** Plasmids used in this study

Name	Origin	Comments
pDN18	Lab collection	Constructed using pCR8/GW/TOPO vector (Invitrogen) and used in the current study as a vector carrying the gentamicin resistance cassette
pES1	Current study	Constructed using DN18 for *tdaB* gene replacement with the gentamicin resistance cassette

### Bacterial growth in monocultures

Bacteria were grown in a 96-well sterile plate in CNS medium with 5.5 mM succinate as the sole carbon source. An initial inoculum of OD_600_ = 0.01 was introduced into 150 µL medium in each well, overlaid with 50  µL of hexadecane (Thermo Fisher Scientific) to prevent evaporation. Growth was monitored at 30°C using the Infinite 200 Pro M Plex plate reader (Tecan Group), and measurements were performed each hour after shaking.

To transform the obtained absorbance values into cells per milliliter, the absorption values were converted to OD_600_ values by multiplying by a conversion factor of 3.8334 (for *P. inhibens*) and 3.1435 (for *D. shibae*) as calculated based on standard curves (Fig. S4). Briefly, standard curves were generated by twofold serial dilution of bacterial cultures prepared as described in Strains and Growth Conditions. For each dilution, plate reader, OD_600_, and CFU per milliliter values were generated and correlated using a linear fit.

Then, values were transformed to cells per milliliter using the equations obtained for the linear fit of OD_600_ and CFU per milliliter values for each strain (CFU = 6.31e + 07 × OD_600_ + 8.35e + 05 for *P. inhibens*; and CFU = 4.11e + 08 × OD_600_ + 1.05e + 07 for *D. shibae*).

### TDA susceptibility plate assay

Liquid pre-cultures of *D. shibae*, *P. inhibens* WT, and *P. inhibens* Δ*tdaB* were prepared as described earlier. *D. shibae* was plated using sterile cotton swabs on MB agar plates to create a lawn. The lawn was dried, and on top of it, 5 µL of *P. inhibens* WT or *P. inhibens* Δ*tdaB* cultures (in duplicate) was placed and allowed to dry. Plates were incubated for 5 days at 30°C, allowing visible colonies of *P. inhibens* WT or Δ*tdaB* to develop. The pigmentation and the presence of an inhibition zone around the *P. inhibens* WT or Δ*tdaB* colonies were evaluated.

### Co- and tri-culture preparation

Bacteria were grown in pre-cultures as described earlier. For co-cultures, bacteria were inoculated in 10 mL CNS medium with 5.5 mM succinate as the sole carbon source in a final concentration of OD_600_ = 0.005 calculated for each bacterial strain (OD_600_ of pre-cultures was measured with spectrophotometer Ultrospec 2100 Pro, Biochron). Co-cultures were cultivated in 30°C in the dark with shaking (130 rpm).

For tri-cultures, algal cells were inoculated in ASW with L1-Si supplements (250 mL Erlenmeyer flasks) in a final concentration of 10,000 cells/30 mL of medium. Using sequencing, we have previously established that the combination of observation under the microscope and plating indeed indicates the axenic state of the cultures (1). Four days later, bacteria from liquid pre-cultures were diluted in sterile ASW and added to algae to a final concentration of no more than 50 cells/mL for each strain (bacterial concentrations in pre-cultures were estimated by OD_600_ measurements). The final concentration of bacterial cells was confirmed by colony counts on agar plates (as described below). Cultures were kept in 18°C under a light/dark cycle of 16 h/8 h (130 µmol photons/m^2^/s) without shaking. Each time a flask was mixed for sampling, it was eliminated from the experiment.

### Monitoring bacterial growth in co- and tri-cultures

#### Bacterial enumeration via CFU

To estimate bacterial cell concentrations in the cultures, colonies were counted on selective agar plates. As *D. shibae* growth is inhibited when plated together with *P. inhibens* WT on one-half YTSS agar plates, selective plates were developed (one-half MB with 10 µg/mL kanamycin) for counting *D. shibae*. On these selective plates, *D. shibae* can grow, but *P. inhibens* cannot grow. For CFU counts of the various cultures, 10 µL of diluted cultures was plated on agar plates containing one-half YTSS to count *P. inhibens*, one-half YTSS with 30 µg/mL gentamicin to count *P. inhibens* Δ*tdaB*, and one-half MB with 10 µg/mL kanamycin to count *D. shibae*. After 2 days for *P. inhibens* and 5 days for *D. shibae*, colonies were counted, and a CFU per milliliter value was calculated based on the dilution factor. Two independent technical repeats on separate agar plates per each biological replicate were performed.

#### Bacterial enumeration via qPCR analysis

Bacteria are known to form aggregates when cultivated with algae (2), and this might affect the results of CFU counts on agar plates. Therefore, to validate the bacterial counts in tri-cultures, a qPCR method based on DNA copy number was used as an independent enumeration method.

For this, 20 mL of tri-cultures after 8 days of cultivation (in triplicate) was pelleted by centrifugation at 13,000 × *g* for 2 min at 4°C. Samples were kept on ice at all times to prevent DNA degradation, and the pellets were kept at −20°C until DNA extraction. Genomic DNA was extracted using Wizard Genomic DNA Purification Kit (Promega, USA) following the manufacturer protocol for bacterial DNA extraction. The final concentrations of extracted DNA were measured with Quibit HS dsDNA assay (Thermo Fisher Scientific).

A species-specific set of primers was designed for each bacterial species using Mauve software (46), BioCyc (47), and National Center for Biotechnology Information (48) databases (Table 4). The product size was 130 bp for all primer pairs. A calibration qPCR confirmed no off-target amplification with both bacterial and algal genomes, for each pair of primers (Fig. S6).

**TABLE 4:** Primers used in this study for qPCR and bacterial genome length

Strain	Forward unique primer	Reverse unique primer	Genome length (bp)
*Phaeobacter inhibens* DSM 17395	TGTGCGTTCAAGGTAGACCA (#1071)	CGACCTTTTCGTTCGTTCAA (#1085)	4,227,134
*Dinoroseobacter shibae* DFL-12	TCTCACACCCTATCCGCTGG (#1067)	GTCGAAGTTCTCCGAAGGGT (#1068)	4,417,868

qPCR was conducted in 384-well plates, using SYBR Green Master mix (Thermo Fisher Scientific) in a QuantStudio 5 (384-well plate) qPCR cycler (Applied Biosystems, Foster City, USA). The qPCR program ran according to the enzyme requirements for 30 cycles (annealing temperature 60°C). Primer efficiencies and standard curves were obtained by qPCR amplification of known DNA concentrations (Fig. S5) and analyzed using QuantumStudioTM Design & Analysis Software (Thermo Fisher Scientific). The results of the experiments were analyzed using the obtained standard curves.

To convert nanograms of DNA into copy numbers of bacterial genomes, the following formula was used, as previously reported (12):


$$DNA\;copy\;number=\frac{DNA\;concentration\times 6.022\times 10^{23}}{Genome\;length^{*}\times 10^{9}\;\times 650\;}$$

where 6.022 × 10^23^ is Avogadro’s number, 10^9^ is the conversion from gram to n nanogram, 650 is the average weight of a base pair (g/mole), and * is the genome length of bacterial strains (listed in Table 4).

Of note, qPCR and CFU counts are complementary but not directly correlated methods. While CFU counts can underestimate bacterial numbers due to attachment and aggregation, qPCR may overestimate them by detecting DNA from dead cells, making the two methods complementary. Additionally, qPCR analysis is affected by genomic DNA loss during the DNA extraction process.

### Monitoring algal growth

Algal concentrations in cultures were measured by a CellStream CS-100496 flow cytometer (Merck, Darmstadt, Germany), using 561 nm excitation and 702 nm emission wavelength. All algal samples included three biological replicates. The only exception was the data point for algal growth on day 9 (Fig. 4C), which included two biological replicates due to a technical sample loss. For each sample, 50,000 events were recorded (with the exception of day 0), and all measurements were performed using technical duplicates. For day 0, due to the dilute nature of the culture, 1,000 events were recorded, and the average value of three biological replicates, before bacterial addition, was calculated. Algal cells were gated according to event size and fluorescence intensity.

### TDA measurement

To quantify extracellular TDA concentrations, mono-, co-, and tri-cultures were prepared as described previously in 30 mL culture volume, with four biological replicates. After reaching the desired time point (as noted in Table 1), cultures were filtered using a vacuum filtration system with a Milipore Express PLUS Membrane (0.22 µm) (Merck).

Based on the brown color of the cultures, which is indicative of TDA production (16), two approaches were employed to process samples with high and low expected levels of TDA. Samples with dark brown coloring and a high expected concentration of TDA (such as mono- and co-cultures after 24 h of incubation) were treated based on a previously published protocol (49) with the following changes. The pH of the samples was adjusted to roughly 3 by adding 30 µL acetic acid to 1 mL sample, centrifuged and transferred to vials. Samples with light brown coloring and a low expected concentration of TDA (such as tri-cultures after 4 and 7 days of cultivation) were brought to pH 3 by adding 3% acetic acid, and the samples were applied on Phenomenex StrataX 33 µm polymeric reversed phase 1 mL/30 cc cartridges, preconditioned with 1 mL ACN, 1 mL methanol (MeOH), and 1 mL 3% acetic acid. After loading the samples (23–27 mL), the cartridges were washed with 1 mL 3% acetic acid and eluted with 1 mL 3% acetic acid in MeOH. The eluents were evaporated to dryness under a gentle nitrogen stream and reconstituted in 50 µL 1:1 mixture of MeOH:double-distilled water (DDW) acidified with 3% acetic acid.

TDA was measured using a UPLC ACQUITY (Waters Corp., Massachusetts, USA) system coupled to a TQ-XS mass spectrometer (Waters Corp.). The liquid chromatography method was performed as described previously (50) for semipolar compounds using an ACQUITY UPLC BEH C18 column (2.1 × 100 mm, i.d., 1.7 µm) (Waters Corp.). Mobile phase A consisted of 5% ACN in DDW, while mobile phase B was 100% ACN, both supplemented with 0.1% formic acid. The column was maintained at 35°C; the flow rate of the mobile phase was 0.3 mL/min. Mobile phase A was initially run at 100% and was gradually reduced to 72% at 22 min, following a decrease to 60% at 22.5 min and then a decrease to 0% at 23 min. Then, mobile phase B was run at 100% until 26 min; mobile phase A was then set to 100% at 26.5 min. Finally, the column was equilibrated at 100% mobile phase A until 28 min. The capillary voltage was set to 1 kV at positive ionization mode; cone voltage was set to 20 V. The measurement was performed in MRM mode. The transition 212.8 > 150.8 (collision energy 22 eV) was used for quantification. The following transitions were used for identification: 212.8 > 150.8 (collision energy 22 eV), 212.8 > 166.8 (collision energy 20 eV), 212.8 > 82.8 (collision energy 27 eV), 212.8 > 110.8 (collision energy 25 eV), and 212.8 > 138.8 (collision energy 25 eV). The retention time of TDA was 11.39 min. Data were processed with MassLynx software with Targetlynx (Waters). Quantification of samples that were analyzed without further processing, such as mono- and co-cultures after 24 h of incubation, was performed directly using an external calibration curve prepared in a pool of Δ*tdaB* monoculture samples acidified with 3% acetic acid. Analysis of samples that underwent solid-phase extraction (SPE) was performed using a calibration curve prepared in a 1:1 mixture of MeOH:DDW acidified with 3% acetic acid. The limit of detection (LOD) in the UPLC vial was 1 ng/mL, which corresponds to an estimated LOD of 8 pg/mL in the original sample, based on a 500-fold concentration factor and an assumed 25% recovery rate during SPE.

### Statistics

At least three biological replicates were used for each treatment. For each biological replicate, the mean of at least two technical repeats was calculated. Statistical analysis included two-tailed *t*-test and was performed using Spyder 6.0.1 (Python Software Foundation).

## Data Availability

All data are available in the article or supplemental information. Plasmids and bacterial strains generated in this study will be made available on reasonable request.

## SUPPLEMENTAL MATERIAL

Supplemental Figures msphere.00138-25-s0001.pdf Figures S1-S6.

ASM does not own the copyrights to Supplemental Material that may be linked to, or accessed through, an article. The authors have granted ASM a non-exclusive, world-wide license to publish the Supplemental Material files. Please contact the corresponding author directly for reuse.
