# Supplementary material for: The impact of tropodithietic acid on microbial physiology under varying culture complexities
Source: mSphere. 2025 Jul 18;10(8):e00138-25. doi: 10.1128/msphere.00138-25 (PMC12379602; doi:10.1128/msphere.00138-25)
Supplement: Supplemental Figures — Figures S1-S6. [file msphere.00138-25-s0001.pdf]

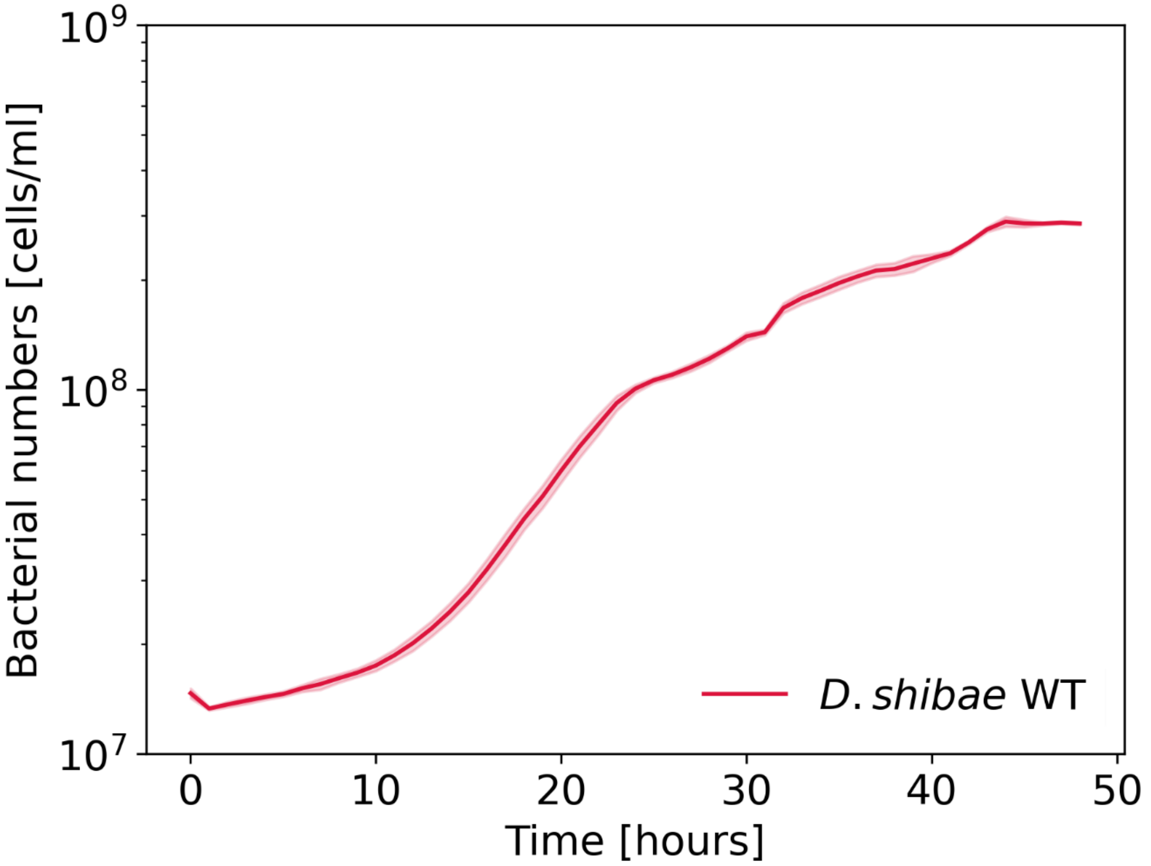

Figure S1. Growth of *D. shibae* bacteria in mono-culture*.* Bacteria were grown in CNS medium containing succinate as a sole carbon source. Bacterial growth was monitored using OD_{600} and values were converted to cells/ml (see Materials and Methods). The line represents the mean of four biological replicates, and the shaded area indicates the standard deviation.

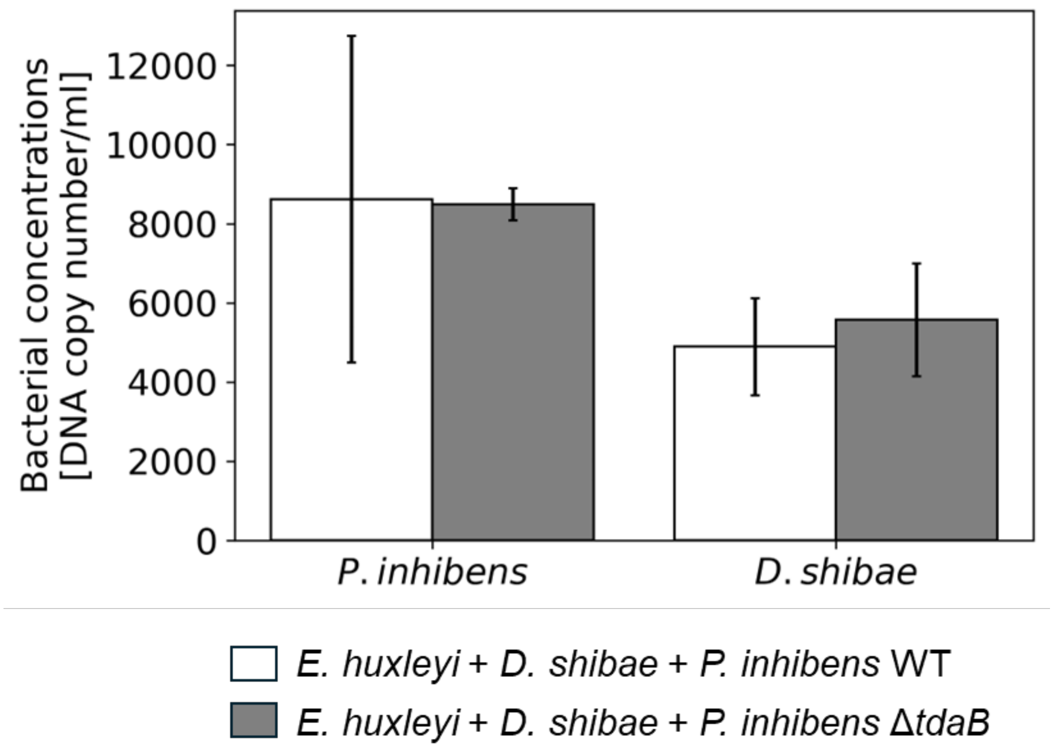

Figure S2. The deletion of the *tdaB* gene does not affect bacterial growth in tri-cultures. Bacterial DNA copy numbers on day 8 in tri-cultures with *P. inhibens* WT (white bars) or Δ*tdaB* mutant (grey bars) measured by qPCR (see Materials and Methods). Genomic DNA was extracted from cultures before algal death was evident. Bars represent mean values of three biological replicates, error bars indicate the standard deviation.

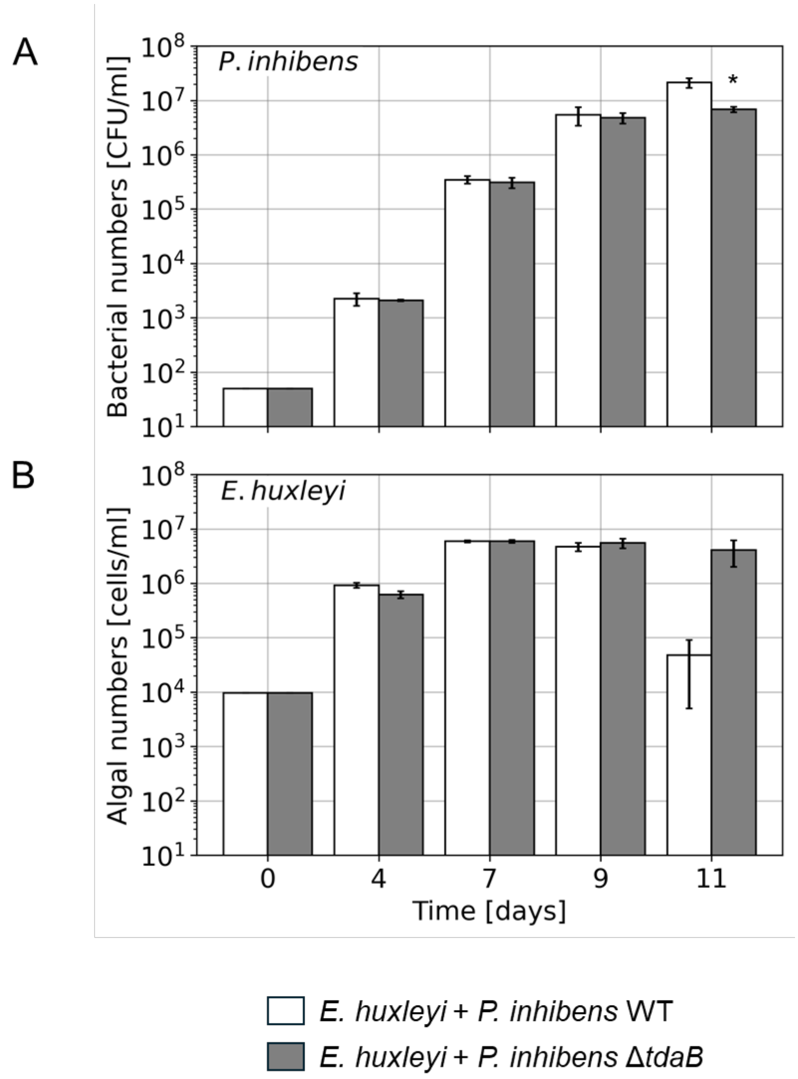

Figure S3. The deletion of the *tdaB* gene delayed the pathogenicity of *P. inhibens* towards the algal host also in cultures without *D. shibae*. A *P. inhibens* WT (white bars) and Δ*tdaB* mutant (grey bars) growth in cultures with algal host **B** Algal host *E. huxleyi* growth in cultures with *P. inhibens* WT (white bars) and Δ*tdaB* mutant (grey bars). Bacterial growth was monitored using selective plates, and algal growth was tracked using flow cytometry (see Materials and Methods). The differences in growth between cultures were evident at day 11 for *P. inhibens*. Bars

represent mean values of three biological replicates, error bars indicate standard errors. * indicates $p<0.05$ using a two-tailed t-test on log-transformed values.

Importantly, the delay in algal death was observed in cultures with the *tdaB* mutant strain compared to cultures with the WT. However, the difference in algal numbers on day 11 (lower panel) between cultures with WT versus the *tdaB* mutant did not yield statistically significant differences. Notably, the variability observed at the onset of algal death, coupled with limited temporal resolution (sampling every two days), likely contributed to the variability observed on day 11. Excluding one replicate (which exhibited earlier death) does yield statistically significant results. However, we consider the observed variability to be an inherent characteristic of this system and have therefore chosen to present the complete dataset, reflecting this variability.

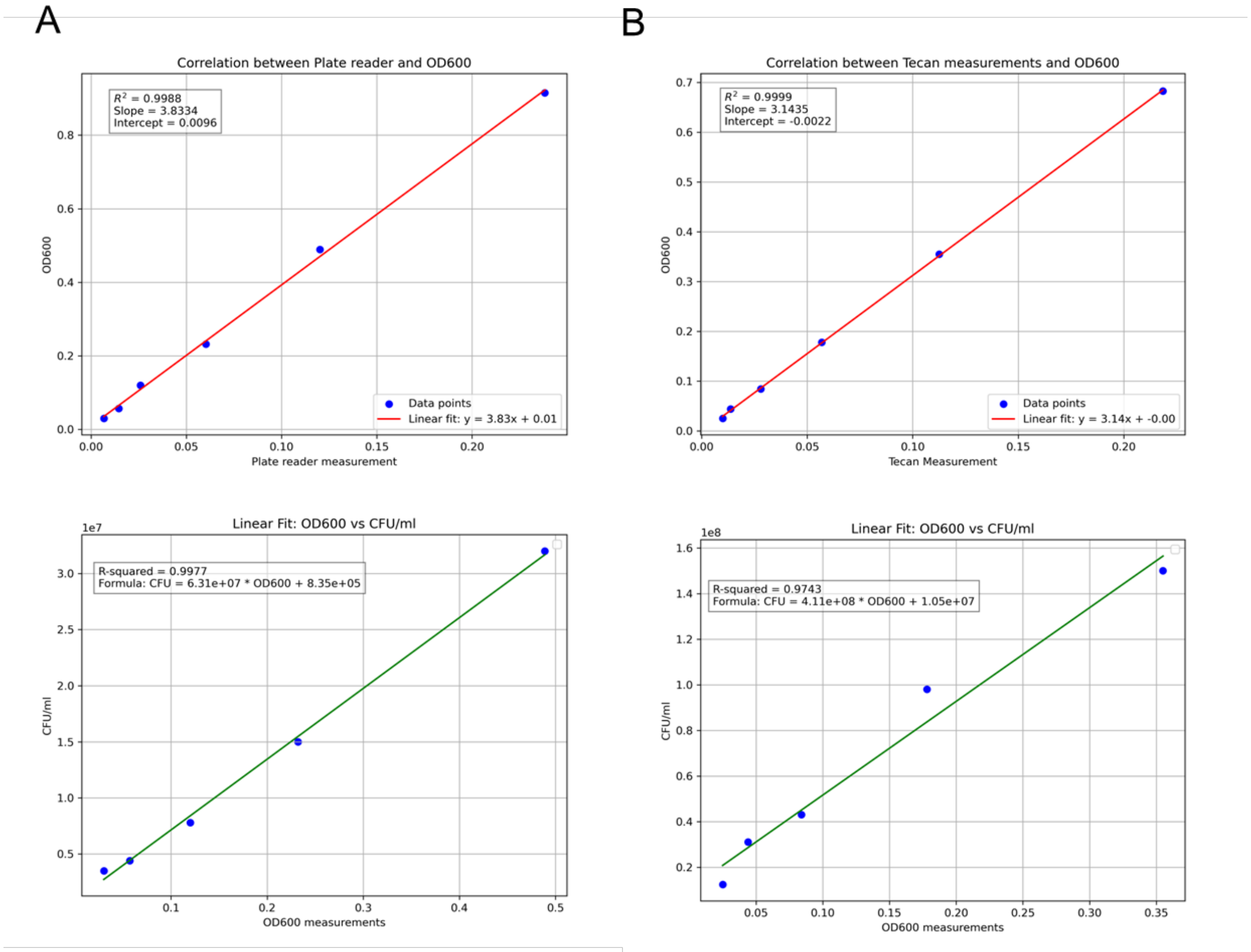

Figure S4. Standard curves for conversion of plate reader values to OD_{600} and CFU/ml. Standard curves were generated for *P. inhibens.* (A) and *D. shibae* (B) bacteria, for conversion of plate reader values to OD_{600} (upper panels) and OD_{600} values to CFU/ml (bottom panels). Samples were generated using 2-fold serial dilutions of bacterial cultures in ASW.

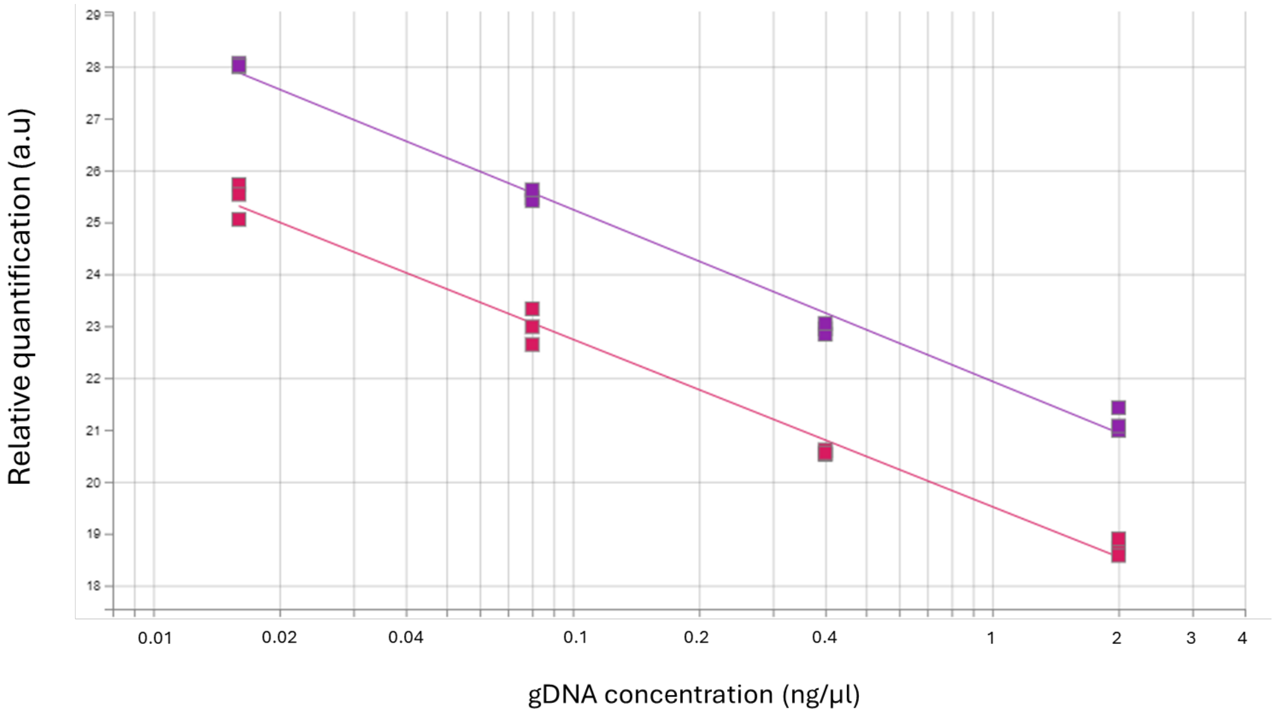

Figure S5. Standard curves for qPCR species-specific primers. Standard curves were generated using 2 ng/µl of the target gDNA as the initial concentration. The initial concentration was serially diluted using a 1:5 dilution factor to generate a total of 4 dilutions. For each dilution, three technical replicates were used. Amplification was performed using primers 1071-1085 (specific for *P. inhibens,* pink) and 1067-1068 (specific for *D. shibae,* violet). Primer efficiencies are 104.2% for primers 1071-1085 (R^2 = 0.989) and 100.5% for primers 1067-1068 (R^2 = 0.992).

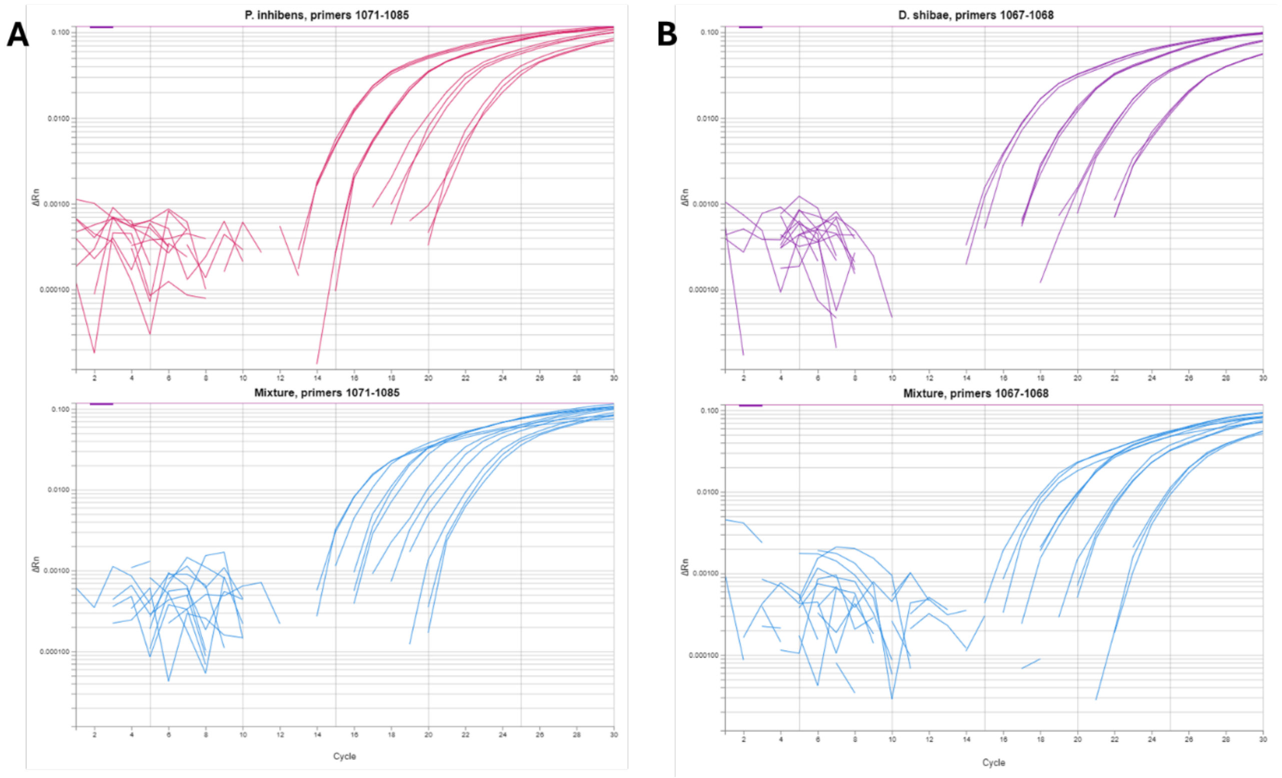

Figure S6. Amplification plots of bacterial gDNA in samples containing either single species gDNA or a mixture with non-target DNA, using species-specific primers. Serial dilution of gDNA extracted from *P. inhibens* (A, upper panel) and *D. shibae* (B, upper panel) were used to test the specificity of primers. A mixture containing gDNA from *P. inhibens*, *D. shibae* and *E. huxleyi* was combined in a 1:1:1 ratio and used as template for amplification with species-specific primers (lower panels). The starting concentration of the target gDNA was 2 ng/µl (both in the one-species and mixture samples) and it was serially diluted using a 1:5 dilution factor. Amplification was performed using *P. inhibens* (A) and *D. shibae* (B) species-specific primers.
